# The Pivotal Role of Quality Technical Structures for Clinical Trials Oversight in the Achievement of Long-Term Capacity Strengthening Outcomes

**DOI:** 10.3389/fmed.2022.772605

**Published:** 2022-03-02

**Authors:** Solomon Owusu Sekyere, Ivana Škrnjug-Yudov, Alexander Pinz, Heidi Meyer, Christoph Conrad

**Affiliations:** ^1^Paul-Ehrlich-Institut, Federal Institute for Vaccines and Biomedicines, Langen, Germany; ^2^RegTrain-VaccTrain of the Global Health Protection Programme (GHPP), Paul-Ehrlich-Institut, Langen, Germany; ^3^WHO Collaborating Centre for the Standardization and Evaluation of Vaccines, Paul-Ehrlich-Institut, Langen, Germany

**Keywords:** staff training, capacity building, clinical trials, clinical trials oversight, training outcomes, RCORE training fellowship, medicines regulation, regulatory systems strengthening

## Abstract

**Background:**

Development of safe and efficacious medicines in many sub-Sahara African countries remains hampered due to fragmented health research infrastructure and ineffective regulatory oversight. To boost the latter in the area of Clinical Trials (CT) Oversight (CTO), many international programs and Regional Centers for Regulatory Excellence (RCORE) initiatives offer various trainings to help strengthen human resource capacity. Here, we aimed at evaluating the training outcomes (at home-institution level) of sponsored fellows for one of such capacity strengthening interventions; a measure that is less often reported and thus remains poorly understood.

**Method:**

The Global Health Protection Programme's VaccTrain project sponsored nine regulatory staff from eight National Medicines Regulatory Authorities (NMRAs) in sub-Saharan Africa for the RCORE CT Training Fellowship by FDA Ghana in a particular year. Using a systematized evaluation framework based on the theory of change, we assessed the individual- and NMRA-level achievement of pre-defined training outcomes. For this purpose, data was collected at pre-training and at short- and long-term evaluation time-points using a survey instrument.

**Results:**

At pre-training, our data revealed existence of differential expectations and orientations among the training participants, thus providing an early indication of potential distinctive patterns in achievement of desired training outcomes. In a short-term post-training follow-up evaluation, a two-group clustering of fellows based on the achievement of training outcomes where only one group (representing 44%) reported achievement of CTO-related outcomes was observed. At this time-point, achievement of training outcomes was associated with the vibrancy of CT activity and existence of a comprehensive technical structure for CTO. In a further long-term follow-up evaluation, our data revealed a successful achievement of CTO-related individual- and/or institutional-level outcomes in all but one fellow. Here again, availability of a robust technical structure for CTO (and perhaps fellow affiliation/selection)–but not CT vibrancy–showed a trend of temporal association with achievement of training outcomes.

**Conclusion:**

Given the pivotal role operational structures of international standards at home institutions play in translating training-acquired knowledge into measurable CTO-related outcomes, we encourage that capacity strengthening projects aimed at achieving health-related targets of Sustainable Development Goals adopt an approach built on this foundation.

## Introduction

The current global migration of clinical research and shifts toward disease endemic regions has resulted in Africa becoming an important destination for many product developers across the globe to conduct clinical trials (CTs) (1–4). In order to efficiently deal with this upsurge and ensure safety of research participants and scientific integrity of clinical data, a commensurate rise in strong and effective functional research oversight is warranted. In many countries on the continent, however, regulatory systems and structures for such activities are often not readily available, weak, or of limited capacity (5–8). This was particularly exemplified by the issues of CTs for vaccine candidates during the Ebola virus disease outbreak in West and Central Africa (9) and in the currently on-going covid-19 pandemic (10). It was for this reason that the current Partnership for African Vaccine Manufacturing (PAVM) initiative by African Union and Africa CDC considers regulatory systems strengthening as one of the cornerstones to their efforts aimed at accelerating Africa's involvement in the clinical development of vaccines to ensure its timeous and unfettered access on the continent (11, 12). To help boost regulatory capacity of many National Medicines Regulatory Authorities (NMRAs) in the sub-region, substantial investments have been made in different priority areas by different international and pan-African institutions using various capacity strengthening strategies (13–16). For Clinical Trial Oversight (CTO), staff scientific and regulatory training mediated through various international programmes or by the African Union Development Agency—New Partnership for Africa's Development (AUDA-NEPAD)-designated Regional Centers of Regulatory Excellence (RCOREs) (17) have often been the norm. Thus far, numerous of such trainings have been provided to staff of various NMRAs in the past years (16, 18–22). However, only little published data is available on robust evaluation modalities of their effectiveness in practice and the specific outcomes of training activities at the level of the local NMRAs (23).

One of the models proposed as a conceptual framework for mapping capacity and evaluating the effectiveness of interventions was developed over four decades ago (24). This logic model, as recently adapted by PHINEO (25, 26) and also employed by United Nations Development Programme (27) and the World Health Organization (WHO) (28), is based on the theory of change and defined by a chain of events comprising inputs—outputs—outcomes—and impact of a project's operations. Thus, this framework draws on a simplified, systematic cause-effect scheme where focus is maintained on the interconnections and dependencies that exist between the various components in achieving pre-defined desired outcomes and impact, and not just on the amount of input (resources) invested.

At each step on the results chain, respective performance indicators are defined and assessed to monitor the progress and success of the capacity strengthening intervention in a more tangible manner. In this report, we employed a strategy based on this model to monitor effectiveness of our capacity strengthening intervention mediated through the staff training sponsorship for selected CT regulators in sub-Saharan Africa. This intervention forms part of the three-pronged approach to capacity strengthening of our VaccTrain CT project.

VaccTrain is a pilot project of the Paul-Ehrlich-Institut—The German Federal Institute for Vaccines and Biomedicines—conducted under the aegis of the Global Health Protection Programme (GHPP) of the German Federal Ministry of Health. The pilot project focused on regulatory capacity strengthening in the area of CTs in sub-Saharan Africa. Direct target groups of the VaccTrain are CT regulatory staff of NMRAs in sub-Saharan Africa. The project aims at impacting the society of partner countries in ensuring improved access to medicines and novel treatments. In 2019, VaccTrain sponsored the participation of nine NMRA staff from eight countries in sub-Saharan Africa in the four-week RCORE CT fellowship training in Ghana.

Here we present a study where we investigated the short- and long-term outcomes of the sponsored training at the level of individual NMRAs, as part of our monitoring and evaluation protocol. The results of our follow-up analysis revealed important insights into what might be considered as determinants of an effective capacity strengthening intervention. It also provides a learning handle for shaping the strategic planning of sponsorship programmes that seek to improve outcomes in recipient NMRAs.

## Materials and Methods

### VaccTrains's Training Programme for Clinical Trials Regulatory Staff

As part of the comprehensive capacity development agenda of the GHPP-VaccTrain, we utilize external training of CT regulatory staff as an important strategy to translate our technical support efforts into improved scientific and regulatory capacity in CTO at the partner NMRAs. The GHPP VaccTrain project has been providing financial and technical support to the CT RCORE in Ghana Food and Drugs Authority (FDA) since its inception in 2017. In the year for which this evaluation was performed, the sponsorship package was extended to cover 9 fellows (from 8 countries) to partake in the training programme by the FDA Ghana in Accra. The sponsorship was arranged upon agreement between the GHPP-VaccTrain and the CT RCORE of FDA Ghana. Fellows were selected solely by an instituted Ghana RCORE CT committee based on their own set criteria. The training was conducted in English, and all study participants were from countries where English is spoken as an official language.

### Monitoring and Evaluation of Training

To monitor and evaluate progress of this outcome-oriented scientific and regulatory training at the recipient institution's level over time, specific components of the logic model (input, output, outcome, impact) and their respective performance metrics were defined based on PHINEO's comprehensive and prospective evaluation framework (Table 1).

**Table 1:** Overview of the training's evaluation framework-matrix in achieving its set outcomes.

**Logic chain component**	**Definition**	**Case—RCORE training**
Input	All resources (e.g., financial, human, material, etc.) that are used to implement a specific capacity development intervention.	• Financial support/ sponsorship (i.e., financially offsetting the cost of participation in the CT RCORE fellowship)
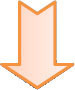		
Output	The direct results of programme or project input activities (e.g., services and products) that are usually relevant to the target group's achievement of outcomes.	• Successful completion of the training by the sponsored fellows according to a curriculum • Trained fellows feeling satisfied with the program and empowered to effect a change for CTO in home NMRA
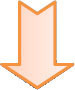		
Outcome	The actual or intended changes in performance or behavior measured in the form of deliverables at the target-group level within a defined period of time	Short-term: • Sharing of the newly-gained knowledge by the sponsored fellows with the colleagues in home NMRA (e.g., internal seminar) • Application of the acquired knowledge and skills in assessment of CTs and regulatory CTO activities at the home NMRA by the sponsored fellows Long-term: • Support to establishment/improvement of NMRA procedures in CTO by the sponsored fellows • Support to establishment /improvement of NMRA procedures for CTO in health emergency situations by the sponsored fellows • Improved competence and efficiency in CT-specific duties at the home NMRA level
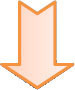		
Impact	The long-term, higher level effect a project is designed to achieve, usually at the societal level	• Improved access to medicines and novel treatments

*A typical case description across the different components of the logic model*.

#### Pre-training Data Collection of Fellows' Expectations

As per our monitoring and evaluation protocol, we first collected pre-training expectations data as a baseline measure to gain a better understanding into the relevance of the training to the selected participants and their respective supervisors. The collected expectations were also to serve as a baseline to track the success of the training in achieving those expectations. A methodology based on a survey instrument comprising a series of descriptive and open-ended (qualitative) questions was developed and shared with the participants 1 week before the start of the training. Specifically, the selected fellows and their home NMRA supervisors were asked to define their specific expectations in the context of their daily challenges (3–5 points). The supervisors were also asked to justify the relevance of the training in the context of current NMRA needs and define expectations that were to be fulfilled by the fellows upon their return from the CT RCORE training.

#### Post-training Data Collection of Fellows' Short-Term Outcomes

Shortly after completion of the training, fellows were reminded and encouraged to keep track of any relative improvements in their day-to-day performance at the NMRA, using their pre-training expectations as baseline. Three months later, a “training report” form was shared with the fellows by the VaccTrain. The fellows were asked to highlight in 3-5 points, as specific as possible (clearly illustrated with examples), the activities they had done in the area of CTs since the training was completed and how the training impacted their daily performance. Their supervisors also were asked to justify the impact of the training based on the tasks performed by the fellows after returning from the CT RCORE training. Feedbacks from the fellows and their supervisors were accordingly analyzed.

#### Post-training Data Collection of Fellows' Long-Term Outcomes

In our monitoring scheme of outcomes of the training at the home NMRA level in the long-term, we collected follow-up data on the individual-level improvements in CT-related duties that were attributable to the training 15 months after the fellowship. In another tier, the NMRA-level improvements in routine CTO and related procedures as well as on CTO procedures in health emergencies was also collected. This was done through an online questionnaire developed using SoSci Survey (29) and made available to participants at *www.soscisurvey.de*.

### Mapping of Training Effectiveness to Parameters That Ensure Successful Implementation and Achievement of Outcomes

Data from the registry and results database ClinicalTrials.gov was further used to aid in mapping country situational reports to the differential dynamics in CTs regulated by the individual NMRAs. The contents of online-available national CT guidelines of respective countries were assessed for their compliance with internationally accepted provisions capable of supporting the documented implementation of newly gained expertise using the WHO Global Benchmarking Tool for CTO (30, 31), similar to a recently reported endeavor for regulatory preparedness in public health emergencies (32).

### Study Cohort

This study included all nine international fellows (from eight countries) who participated in the FDA Ghana RCORE CT Training fellowship under Paul-Ehrlich-Institut's GHPP VaccTrain sponsorship for a particular year. The professional backgrounds of the participants were quite uniform. There were *n* = 7 pharmacists, *n* = 1 medical doctor and *n* = 1 professional nurse. But for two candidates (i.e., F and H) who were reported to be relatively new to medicines regulation, all the other candidates had varying degrees of considerable experience. Overall, the participants either worked in the CT department (*n* = 2), CT and Pharmacovigilance department (*n* = 4), or were affiliated to other departments (*n* = 2). In the latter case, they were either on secondment to the CT department (G) or under co-option for CT duties as per need (E). There was no report on the department of affiliation of one participant. An anonymized list of the sponsored trainees and their expertise/function in their home NMRAs are shown in Table 2.

**Table 2:** Sponsored participants and their respective roles in home institutions.

**Fellow's code**	**Function/expertise at home institution**
A	Clinical trials
B	Clinical trials and pharmacovigilance
C	Clinical trials and pharmacovigilance
D	Clinical trials
E	Medicines registration
F	Clinical trials and pharmacovigilance
G	Testing laboratory
H	Clinical trials and pharmacovigilance
I	Not available

### Ethical Considerations

All participants (respondents) gave their informed consent. Participants were made aware of their reserved right under Articles 17 and 18 of the EU General Data Protection Regulation to request for their data to be erased or restricted in its processing at any time they felt the need to do so. Their right to withdraw their hitherto-granted consent at any time was further highlighted. For ethical reasons, data was processed and anonymously presented to ensure confidentiality. In the same vein, all possible leads that could aid identification of individual fellows were consciously avoided.

## Results

### Assessment of Pre-training Expectations of Selected Fellows

**Figure 1:**
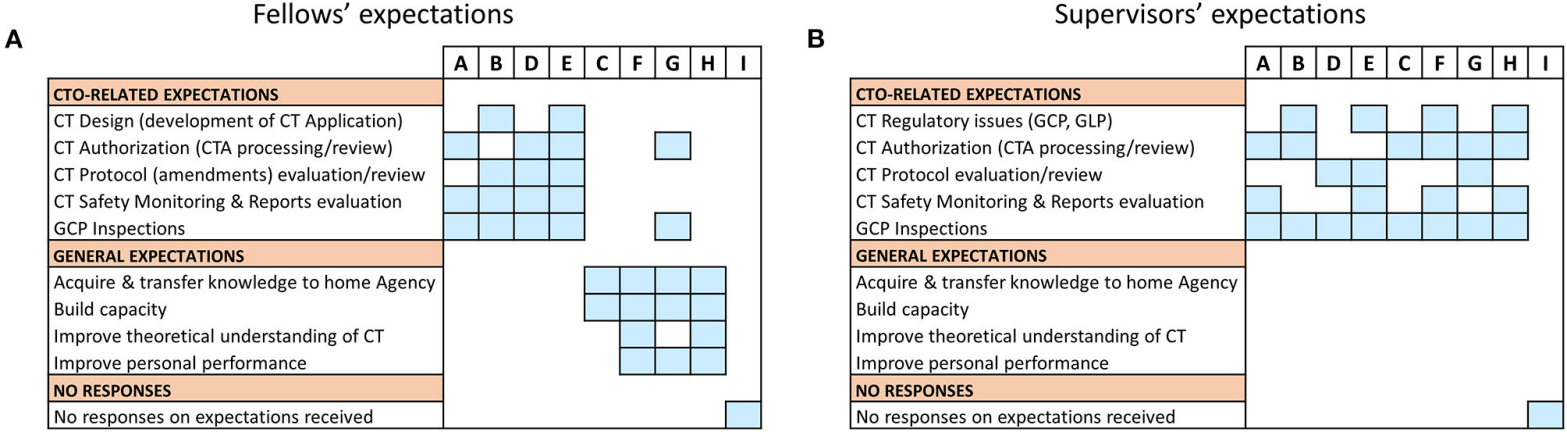
Pre-training expectations of fellows: Data on pre-training expectations were collected one week prior to start of the training fellowship using a questionnaire. **(A)** An individual-based representation of fellows' expectations which were either related to operational Clinical Trials Oversight activities (i.e., CTO-related) or generic and not related to operational Clinical Trials Oversight activities (i.e., non-CTO-related). **(B)** An individual-based representation of supervisors' expectations that were either CTO-related or generic (i.e., non-CTO-related).

To evaluate whether the desired outcomes of our sponsored capacity strengthening intervention was aligned with the expectations of the fellows and their supervisors, the pre-training data on the trainees' expectations was analyzed (Figure 1). In all, eight (8) of nine (9) expected responses were received from the participants and their respective supervisors. There was no response received from candidate I and his/her superior. Overall, a total of 31 individual response points representing an average of 4/person were received. Grouping the participants according to the bearings of their expectations relative to direct CTO activities, we noted they categorized into two main clusters. Whilst one cluster encompassed participants with expectations (*n* = 18) related to operational CT oversight, the other comprised those whose expectations (*n* = 13) were rather generic and non-CTO-related (Figure 1A). Mapping non-CTO-related expectations to specific participants from the different countries, we observed that 11 (representing 84.6%) emanated from the candidates who were either new to CT regulation (i.e., F, H) or on temporary attachment/secondment to their NMRA's CT team (i.e., G). Contrary to the expression of non-CTO-related expectations by a considerable number of the sponsored participants (*n* = 4), all supervisors were clear in a set agenda toward filling specific CTO-related knowledge gaps in their respective NMRAs (Figure 1B). In particular, all supervisors detailed their expected CTO-related deliverables (Figure 1B) and further specified the CTO-related topics that were to be presented by fellows as part of in-house knowledge sharing upon their return (data not shown). Between both the participants and their supervisors, Good Clinical Practice (GCP) inspections and CT authorization (including processing and review) appeared as the topics with high expectations and, perhaps, of particular importance to the fellows. Notably, however, was the observation that significant disparities existed in the participants' expectations and their respective supervisors' (Figures 1A,B). Put together, this data thus revealed the divergent expectations either among the training participants, or between them and their supervisors, thus providing an early indication of potential differential achievement of training outcomes.

### Post-training Evaluation of Short-Term Outcomes at Trained Fellows' Institutions

**Figure 2:**
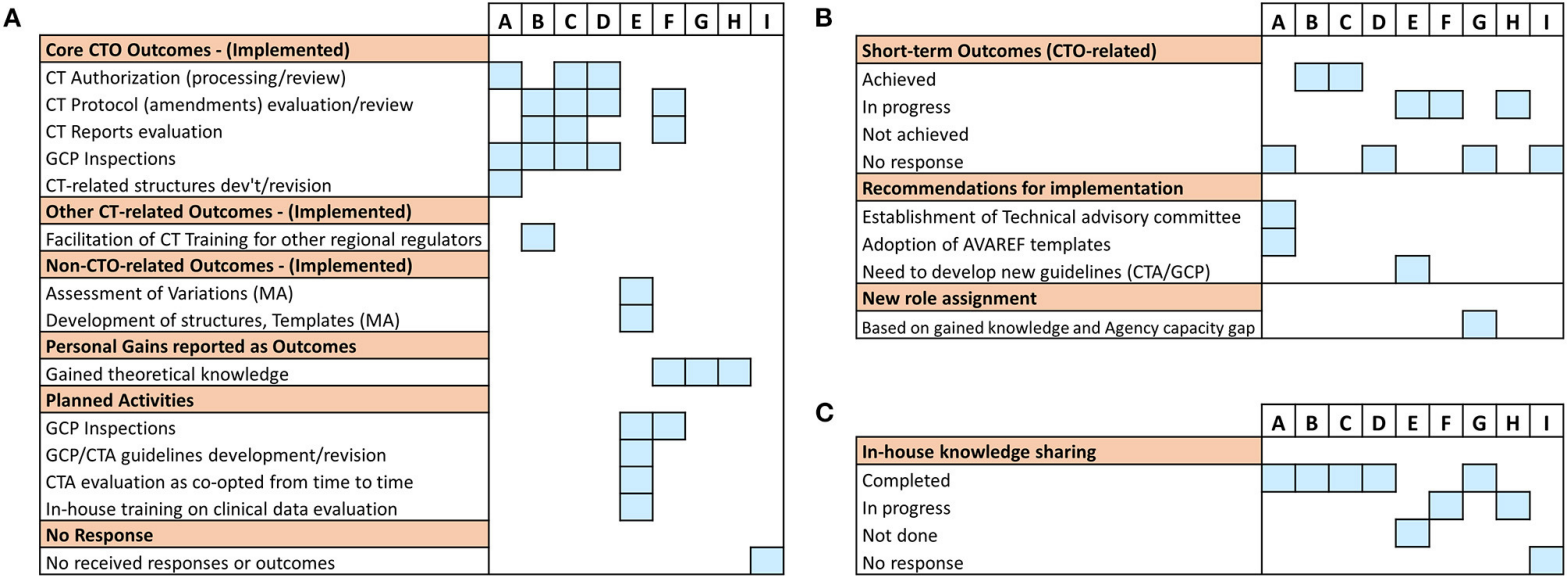
Evaluation of short-term post-training outcomes of the individual fellows: Information was sought either directly from the trained fellows or from their supervisors on issues of achieved training outcomes as well as knowledge transfer 3–4 months after the training. **(A)** A graphical illustration of individual responses from all sponsored fellows showing the kind of reported deliverables (outcomes). **(B)** Individual responses from respective supervisors on the status of post-training outcomes that were expected from the fellows. **(C)** Individual responses from respective supervisors on the status of knowledge transfer or sharing conducted as per NMRA guidelines.

As a next step, we sought to assess the short-term outcomes of the training 3–4 months upon its completion using the predefined framework for mapping (Table 1). Here, we defined outcomes as a measure of deliverables in terms of qualitative and quantitative improvements in daily CTO-related routines of fellows as a result of the training received. As the cumulative analysis of data generated from a disparate cohort like this one consisting of participants with varying expectation profiles has a risk of clouting the representative picture in its truest sense, we resorted to analyzing the participants' outcomes on individual basis (Figure 2A). Similar to the pre-training expectations results, we observed two distinct clusters of responses from the trained fellows based on implementation status of the newly gained knowledge (i.e., achievement of CTO-related outcomes, Figure 2A). While one cluster (fellows A, B, C, and D) reported enhanced performance in CTO activities such as CTA review, CT protocol assessments, CT reports evaluation and GCP inspections, the other cluster (fellows E, F, G, and H) involved responses that only harped on personal theoretical gains in CT regulation, planned activities, and other non-CTO-related outcomes (Figure 2A). We also noted that supervisors' feedback in direct reference to the query on achievement of outcomes further affirmed the trained staff reported outcomes (Figure 2B). Whereas, no supervisor recounted achieved deliverables in the cluster of fellows who reported general knowledge gains, planned activities, or gains relevant to other regulatory functions, two responding supervisors of fellows in the other cluster confirmed enhanced performance in CTO activities (Figure 2B). Interestingly, we further observed that all trained fellows in the cluster that reported CTO-related outcomes had also performed an internal seminar for knowledge transfer contrary to only one fellow in the other cluster who reported a similar achievement (Figure 2C). Our findings not only highlighted a two-group differential achievement of training outcomes, but also revealed lapses in a clear communication of expected outcomes to beneficiaries of external trainings; a scheme that could be useful to ensure in-house sharing and retention of externally gained knowledge.

### Successful Achievement of Short-Term Training Outcomes and Factors That Associate With It

**Figure 3:**
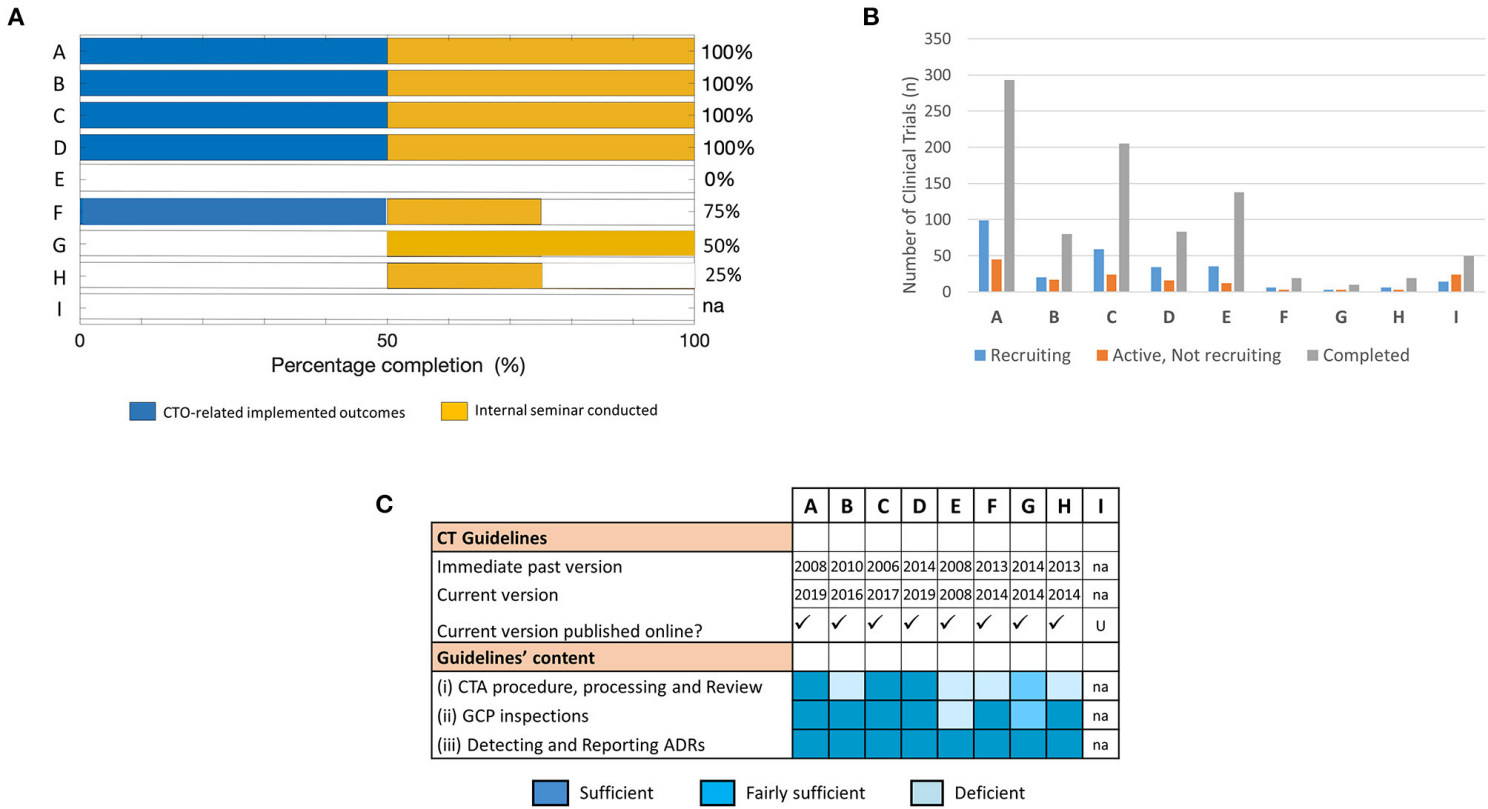
Assessment of training success and factors that influenced achievement of training outcomes at short-term: The effectiveness of the training was mapped to parameters that facilitate implementation success in fellows' NMRAs. **(A)** Percentage completion of short-term project-defined outcomes based on indicators related to implemented CTO-related deliverables and conducted internal seminar for knowledge sharing. **(B)** Number of trial participants involved in CTs at varying stages of recruitment (i.e., recruiting, active—not recruiting, or completed) used as a proxy indicator of CT vibrancy in the respective NMRAs. Data was derived from clinicaltrials.gov on 08.02.2020 (dd.mm.yyyy). **(C)** Availability, accessibility, and content of CT guidelines that govern the conduct of CTs in the jurisdictions of the respective NMRAs of sponsored fellows.

Next, we evaluated the success of the training in achieving the project-defined short-term outcomes using specific predefined indicators as a measure. These specific indicators were formulated on (i) implemented CTO-related deliverables and (ii) facilitated knowledge sharing (e.g., through internal seminars). Using these performance metrics as outlined in Table 1, 4/9 fellows (~44%) reported achievement of project goals with 100% success rate. This was contrary to the 5/9 fellows (~56%) who either did not respond at all or recorded partial or no successfully achieved outcomes (Figure 3A).

We then probed into possible factors that plausibly accounted for the failure to achieve CTO-related outcomes as reported by some of the fellows. Specifically, we utilized data from the registry and results database clinicaltrials.gov to assess proxy indicators of CT vibrancy (such as number of CTs with varying recruitment statuses) in the jurisdictions of the individual NMRAs that may influence achievement of the training goals. Our results show that the cluster of fellows that reported achieved CTO-related outcomes in the short term displayed a higher frequency of completed and ongoing CT activities compared to those that did not (Figure 3B).

CT guidelines are an important component of CT operational structures as they outline regulatory expectations for CTs conducted in a specific country (33–35). We further investigated whether the availability and/or quality of CT operational structures are fundamental to application of training-acquired knowledge to enhance regulatory performance. Thus, we scanned the websites of the individual NMRAs as well as relevant international portals for information on the CT guidelines in the respective countries. All the NMRAs, except that for fellow I, had online-accessible CT guidelines (Figure 3C). Interestingly, however, while the current versions of the CT guidelines of the countries with fellows who reported accomplishment of CTO-related outcomes were relatively new (≤3 years) after having undergone a series of revisions, those available for the other countries were comparatively old (≥5 years) and had virtually not undergone any revision since their initial development (Figure 3C). We further loosely assessed the content of the guidelines as per the precepts of the WHO Global Benchmarking Tool for CTO (30, 31), along the main themes of the pre-training expectations as highlighted in the supplementary information (Tables S1, S2). We observed that only 1 of 4 of the fellows that reported achievement of CTO-related outcomes had significant deficiencies in some parts of their NMRA CT guidelines compared to the other fellows all of whom had significant deficiencies in one or more domains of their available CT guidelines (Figure 3C). There was no observed relationship between achievement of training outcomes and the professional backgrounds of fellows. Put together, these data demonstrated that achieving the desired outcomes of CTO capacity strengthening efforts depended on several factors. Fundamentally, they indicated that implementation of the newly gained knowledge was influenced by the frequency of CTs conducted in that regulatory jurisdiction and depended on the availability and quality of CT operational structures (e.g., guidelines) in the NMRA.

### Long-Term Follow-Up Analysis of Training Outcomes and Its Impact on CTO Activities at the Home NMRA Level

**Figure 4:**
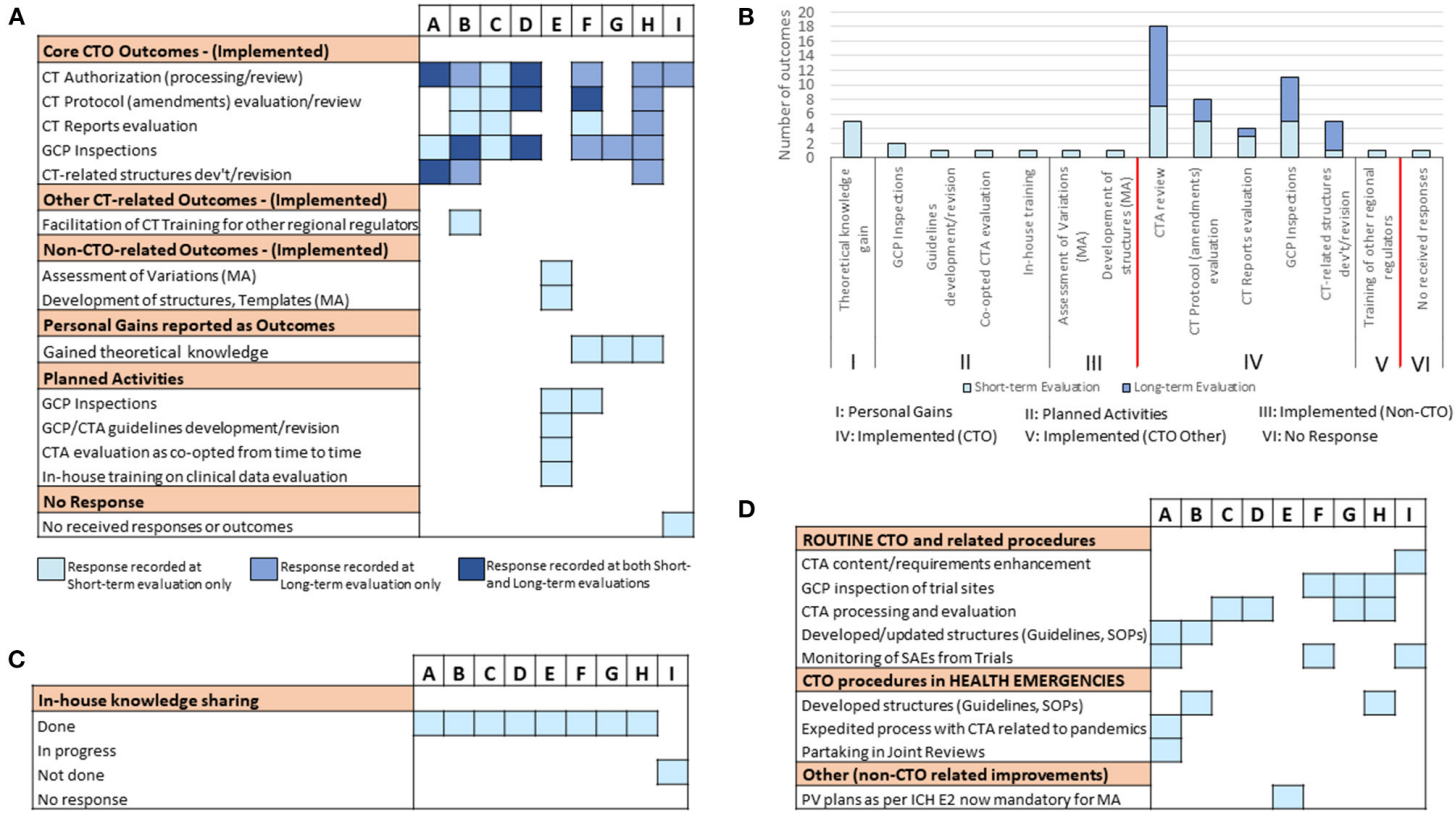
Long-term follow-up evaluation of fellows' outcomes and improvements of CTO activity at the NMRA level: Information was sought from fellows using an electronic survey on issues of individual-level achieved training outcomes and organizational-level improvements in CTO 15 months after the training. **(A)** Individual responses from all sponsored fellows showing the kind of outcomes (CTO-related, non-CTO-related, others) reported cumulatively (i.e., at short- and long-term evaluation time-points). **(B)** A comparative assessment of individual fellow responses (reported outcomes) at the short- and long-term evaluation time-points. **(C)** Individual responses from fellows on the status of knowledge sharing conducted as per NMRA guidelines. **(D)** Individual fellow responses on NMRA-level improvements in routine CTO activities and related procedures as well as CTO procedures in health emergencies.

To follow up on the outcomes of the training in the long-term, and further assess improvements in CTO-related duties at the home NMRA level, a subsequent evaluation was conducted 15 months post-training. The response rate achieved was 100%. The retention rate of fellows at the same Department and in the same role/position as reported before the training was also 100%. We began by investigating whether there had been any new or additional outcomes since the short-term evaluation that could be ascribed to the training-acquired skills of the individual fellows. But for fellows C and E, we observed that all other fellows of the training reported some achieved CTO-related outcomes of a sort at this time-point (Figure S1). The cumulative data (i.e., short-term + long-term evaluations) however revealed fellow E as the only fellow that did not report a single CTO-related outcome 15 months upon completing the training (Figure 4A). It is also worthy of note that unlike the short-term evaluation results, all the outcomes reported in the long-term evaluation were CTO-related (Figures 4A,B). Similar to the short-term evaluation data, CTA processing/review and GCP inspections were the front-running disciplines that registered the most reported outcomes. However, development/revision of CT technical structures (including guidelines, standard operating procedures, Advisory Committee's Terms of Reference, etc.) was the CTO discipline that witnessed the highest fold increase (i.e., five-fold) in the number of outcomes from the short-term to the long-term time-points (Figure 4B). The evaluation also revealed that all the fellows, except for fellow I, had conducted an internal seminar for knowledge transfer at this time-point in their respective NMRAs (Figure 4C). Further to these investigations at the individual level, we next probed the institutional level for CT procedures that had been improved as a result of the training-acquired expertise of the respective fellows (Figure 4D). We observed that all fellows, but one, reported outcomes at this level in routine CTO and related procedures and/or CTO procedures in health emergencies. Fellow E again, the only exception, however, reported an outcome at this level that was rather non-CTO-related (Figure 4D). In all, these data suggested that there are both short- and long-term outcomes that may be attributed to the training thus making it a sustainable intervention.

### Successful Achievement of Long-Term Training Outcomes and Factors That Associate With It

**Figure 5:**
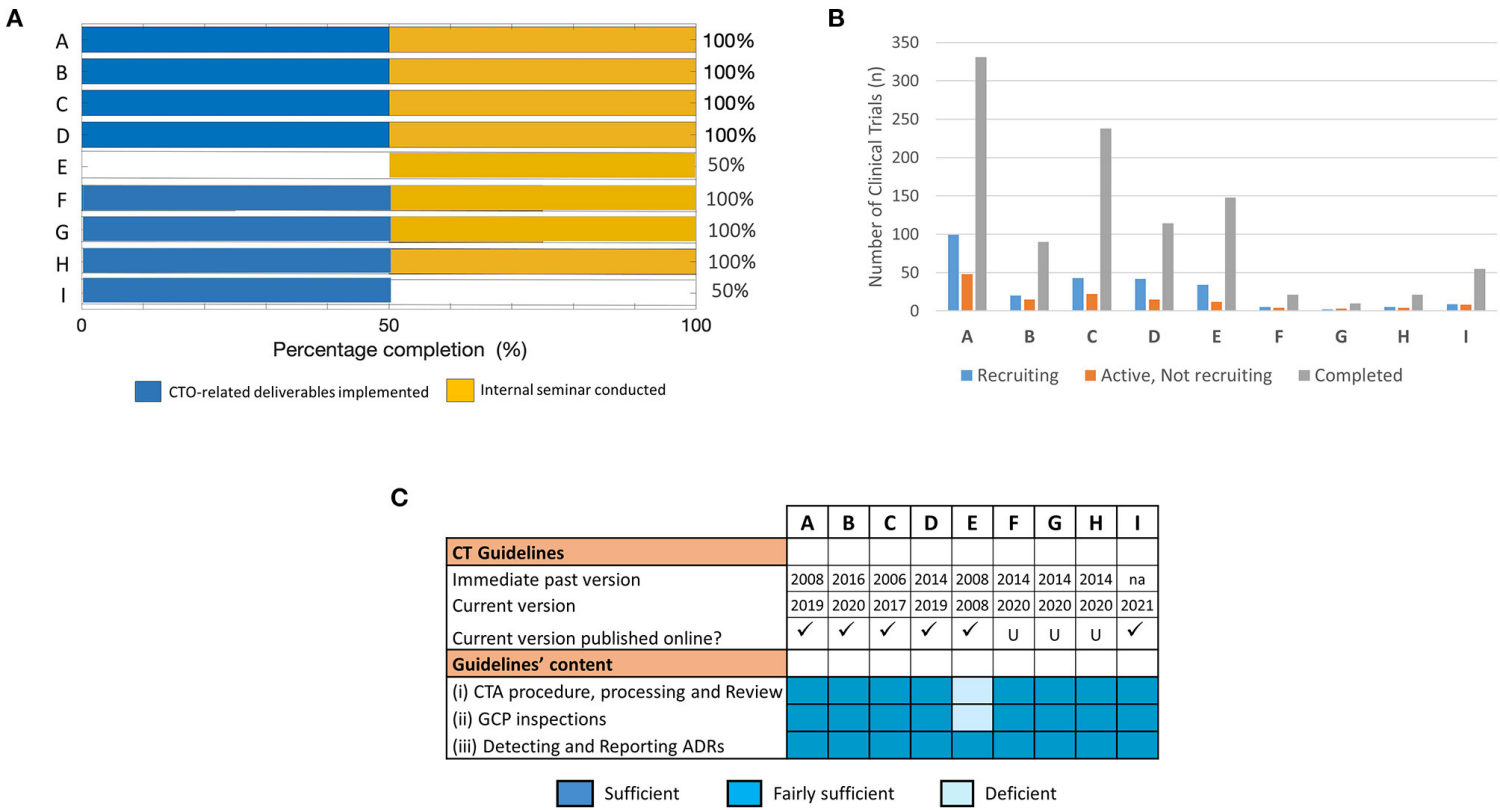
Assessment of training success and factors that influenced achievement of training outcomes at long-term. The effectiveness of the training was mapped to parameters that facilitate implementation success in fellows' NMRAs. **(A)** Percentage completion of long-term project-defined outcomes based on indicators related to implemented CTO-related deliverables and conducted internal seminar for knowledge transfer. **(B)** Number of trial participants involved in CTs at varying stages of recruitment (i.e., recruiting, active—not recruiting, or completed). Data was derived from clinicaltrials.gov on 03.02.2021 (dd.mm.yyyy). **(C)** Availability, accessibility, and content of CT guidelines that govern the conduct of CTs in the jurisdictions of the respective NMRAs of sponsored fellows at the long-term evaluation time-point.

Finally, we aimed at analyzing the status of training outcomes of the individual fellows in the long-term and further assessing the resultant outlook of CT activity and quality technical structures earlier identified as its possible associating factors. First quantifying the success of the training in achieving the project-defined outcomes at this time-point using the predefined metrics, we observed that 7/9 fellows achieved the training-defined goals with 100% success rate (Figure 5A). These included fellows who reported having achieved this feat either early on during the 3–4 months evaluation (i.e., A, B, C, and D) or later at 15 months post-training (i.e., F, G, and H). Of the 2/9 fellows who had not achieve a 100% success rate by the time of our long-term evaluation, 1 (i.e., I) reported clear CTO-related outcomes achieved at this time-point although an internal seminar for knowledge transfer had still not been conducted. Clearly, E remained the only fellow to have not reported any outcomes directly related to operational CTO activities but had performed his/her post-training knowledge transfer duties, albeit only after our short-term evaluation (Figure 5A). Further mapping the dynamism in the achievement of training outcomes to the differential profiles of CT activity in the NMRAs of the respective fellows, we observed no clear association at this time-point (Figure 5B). In fact, the pattern of CT vibrancy in the different NMRAs at this time-point remained similar to that observed during the short-term evaluation. On the contrary, however, we observed what appeared to be a temporal link between achievement of training outcomes and the availability of a robust functional infrastructure (here, operational guidelines) that undergird the conduct of CTs. This was evidenced by the cases of fellows F, G, H, and I all of whom achieved long-term training outcomes in coincidence with revamped CT technical structures (including operational guidelines) through a capacity strengthening exercise (Figure 5C). Indeed, except for the NMRA of fellow E, all the NMRAs of the other 8/9 fellows had CT guidelines that were up-to-date and fulfilled the expectations of the WHO GBT for CTO relative to indicators in the areas of CTA procedure, processing, and review, GCP inspections and monitoring and reporting Adverse Drug Reactions (ADRs, Figure 5C). In summary, the NMRAs that managed to change CT guidelines by the long-term evaluation time-point and improved their quality in a way that aligned with international standards, reported concurrent improvement in training outcomes in a way similar to the NMRAs that already had them. These data therefore highlighted the robust operational CT structural establishment as fundamentally important and playing a central role in translating the RCORE CT training outputs into the intended outcomes at the level of recipient NMRAs.

## Discussion

International support for capacity building is often recognized as a key mechanism for helping many developing countries execute their national plans of achieving the health-related targets of the United Nation's Agenda for Sustainable Development (36). In this regard, many development programmes have made tremendous strides and contributions to strengthening the regulatory capacities of NMRAs in sub-Saharan Africa. Despite these efforts, significant capacity deficit challenges that are detrimental to achieving improved outcomes and impact persist (37). Lack of evidence about the relevance of capacity strengthening interventions is often discussed (38). Prospective monitoring and evaluation of such programmes is deemed as key in ascertaining the value of the investment and ultimately guiding continued funding and sustainability inquests (39). In this study, we drew on PHINEO's systematized four-part capacity building evaluation framework based on the theory of change to systematically assess the short- and long-term effects of our sponsored RCORE CT staff training in achieving a set of intended changes in CT oversight at the level of recipient fellow's NMRAs. Using this tool, which focused on tangible results in a more systematic manner, we here report findings that could be explored in the design and monitoring of similar capacity strengthening interventions to improve implementation success in a more sustainable manner. First, we show that a baseline data collection and evaluation set up is vital in dissecting the differential outcomes of capacity development interventions. Second, our data revealed the immense impact a clear communication between fellows and their respective supervisors could have on the successful achievement of short-term training outcomes, which also associates with CT structures and its vibrancy. Finally, our data demonstrated that selection of the right fellow to be trained and the comprehensiveness of the operational CT technical structure (e.g., CT Guidelines) of an NMRA play a fundamental role in the achievement of the desired long-term outcomes of a CTO capacity strengthening intervention (*such as staff training*).

One cardinal tenet of rolling out an effective capacity strengthening programme is to build on existing local/regional strengths (e.g., expertise, initiatives and institutions) rather than bypassing them (18, 40, 41). In this staff training arm of our tri-component capacity strengthening activities for CT oversight in Africa, we dovetailed our pursuit into the Ghana CT RCORE structure for training regulatory personnel across NMRAs in Africa. This helped us to align our support with that of the Africa Medicines Regulatory Harmonization (AMRH) programme of AUDA-NEPAD. Right from the beginning, specific set of indicators for baseline measures (here called *expectations*), and for assessing intervention effectiveness (or *outcomes*), were developed. This was partly based on the WHO Global Benchmarking Tool for CTO (Table S1) (30, 31), and on our experience in the operational hydraulics of NMRAs of many African countries. Analyzing the baseline data brought to the fore some interesting observations. Particularly amongst them was the considerable number of non-CTO-related expectations reported prior to the start of the training. This mainly involved the new and inexperienced trainees and those who were temporal members of their respective NMRA CT teams. Essentially, the selection of the relatively new regulators by their NMRAs (probably based on their personal knowledge gap and/or that in their CT teams) was a logical one. This was because they stood to benefit from a knowledge gap filling to facilitate their individual- and/or NMRA-level CTO advancement. The selection of the personnel who were not directly involved in day-to-day CTO duties for such training was rather of a concern. This was particularly so because, the compelling nature of the parallel commitments of such trained individuals could contribute to the non-optimal utilization of the training-acquired skills, as was evident following our short-term evaluation exercise. Overall, our pre-training data collection on trainee expectations not only highlighted the fundamental differential expectations of trainees but also provided baseline metrics with which the effectiveness of the training in achieving those pre-training expectations could be tracked.

In the analysis of our post-training short-term evaluation, a two-group clustering which mirrored that displayed by the pre-training expectations data, was evident. While one cluster reported achieved CTO-related outcomes (and largely confirmed by their respective supervisors), the other group of fellows either reported personal gains, planned activities, or gains in other regulatory functions. Again, and interestingly, the latter group of fellows included the two CT newbies and the two staff who were attached to their CT teams on temporary basis. Being new to CT regulation *per se* may not have been a stumbling block in achieving CTO-related outcomes, especially as the training intended output was reportedly achieved (42). The common attribute within the trainees in the latter group therefore became a subject of interest. In aid of this quest, we analyzed the contribution of the academic backgrounds of fellows to the possible variable uptake and translation of training acquired knowledge into measurable outcomes. As the academic and professional backgrounds were quite uniform across the fellows [Pharmacists (*n* = 7); Medical Doctor (*n* = 1); Professional Nurse (*n* = 1)], no such correlation was observed. We further investigated baseline technical structures for CTO in the various NMRAs as well as the vibrancy of CT activity in the respective jurisdictions. First, lack of operational structures of international standards and enabling environments are important factors that could affect the practical utilization of gained knowledge. Practically, the development and implementation of technical structures such as comprehensive guidelines and standard operating procedures provide the basis for structured and systematic operationalization of CTO (and internal knowledge-sharing) activities (43). The absence of these structures would mean that standard procedures are not followed, timelines are unmet, documentation and archiving are not done, and capacity development gains are eroded. Second, it is known that certain so termed ‘environmental factors’ along the entire stretch of the evaluation framework (i.e., from input—impact) influence the effectiveness of capacity strengthening intervention during its implementation phase (44). Particularly in this context is the substantial utility of the vibrancy of CT activity in securing the achievement of regulatory training outcomes in a given NMRA. The availability of the required technical structures and workforce with appreciable capacity in an NMRA would still amount to nothing if there were no feed (i.e., CT application) to trigger the utilization of training-acquired expertise. In our short-term evaluation, we observed a strong association between availability of NMRA's comprehensive CT guidelines and the achievement of CTO-related capacity strengthening outcomes. A similar relationship between availability of NMRA's robust CT guidelines and the conducting of internal seminar for knowledge transfer with the 3–4 month window post-training was also observed. These observations thus made a strong case for CT operational structures of international quality as being fundamentally important in translating training-acquired knowledge into measurable outcomes and efficient knowledge sharing. These data were in consonance with the four-tier hierarchy model of systemic capacity building needs depicted by Potter and Brough, which specifically put structures, systems and roles at the base of the capacity pyramid (43). Further using trial register records from clinicaltrial.gov as a proxy indicator to map the status of CTs conducted within the national borders of the respective NMRAs, we demonstrated that achievement of short-term training outcomes is also associated, at least in part, with the vibrancy of CT activity in each NMRA. An important exception to this particular trend was exhibited by fellow E. Despite established records of viable CT activity in the country of this fellow, s/he did not report any CTO-related short-term outcomes unlike the other fellows with similar CT vibrancy. Of note, fellow E was the candidate who was, but only, a co-opted member of the CT team of the NMRA s/he represented. This observation therefore affirms that right candidate selection and the individual dimension to capacity strengthening are equally key (perhaps as technical structures) to achieving desired short-term outcomes as previously reported (45). This calls for special relevance to be put on endeavors directed at selecting training participants, if attainment of a specific set of short-term post-training outcomes is a programme priority.

Following up on the achievement of the project-expected CTO-related outcomes in a subsequent long-term post-training evaluation exercise, we observed that cumulatively all the fellows but one (i.e., fellow E) reported having achieved a sort of such outcomes. In addition to the successful achievement of these individual-level outcomes, all the fellows also reported improvement(s) in CTO activity(ies) at the institution level. Here too, fellow E was the only exception. Aside its possible relation with the sheer element of time, the achievement of CTO-related outcomes of the training showed a clear association with the CT operational guidelines at the NMRAs. Notably, no such association was observed with the vibrancy of CT activity at this timepoint thus indicating its redundancy and inconsequentiality in the long-term. The sudden availability of comprehensive CT guidelines in the NMRAs of fellows F, G, H and I at the long-term evaluation timepoint became a subject of interest. Probing further into the reason behind it, we discovered that the CT guidelines, together with other regulatory documents like CT regulation and standard operating procedures, were developed through a capacity strengthening support received from an international partner. For us, what was even more interesting was the temporal relationship that seemed to exist between the availability of conventional CT guidelines and the achievement of desired training outcomes. Serving as a pseudo negative control, the only fellow (fellow E) who neither reported any CTO-related individual-level outcome nor an institutional-level improvement of CTO activities also belonged to the only NMRA that had CT guidelines with significant deficiencies at the time of our long-term evaluation. As mentioned before, the primary affiliation of fellow E to another department other than that for Clinical Trials in his/her NMRA was however a noteworthy caveat. On the one hand, this data underscored the fundamental role CT operational structures (and perhaps fellow affiliation/selection) play in the successful achievement of training outcomes. On the other hand, it also illustrated the interdependence of the various levels of capacity needs and the importance of tackling their strengthening holistically (if possible) in order to achieve set results. In the broader scope, this observation awakens the debate on whether or not capacity strengthening interventions should tow the traditional line of further supporting stronger institutions at the detriment of weaker ones. In addition to our position on the utility of multi-component capacity building approaches, we further opine that advancing and leveraging regional and continental networking schemes (like that of the African Vaccine Regulatory Forum, [AVAREF]) as well as fostering various reliance initiatives would be worthwhile in mitigating the negative effects of this skewing tendency.

Our study has obvious strengths and limitations. The major strengths include the (i) prospective nature of the study design and longitudinal collection of data (ii) unbiased nature of the individual-based analysis (iii) long-term follow-up studies of project outcomes and its attendant high response rate and (iv) unique nature of the study cohort that allowed for the temporal correlative studies. The solely descriptive nature of the study, its insufficiency in delineating causal relationships and the fewer number of study participants, however, remain its notable limitations. It will therefore be warranted that further investigation is performed in aid of validating this data in a large prospective-dedicated cohort study or, at least, confirming its reproducibility in a similar study of comparable cohort size. The limitations notwithstanding, this monitoring and evaluation study afforded us the opportunity to amass data which unraveled areas where our intervention failed to occasion the needed change. This information is useful to guide our quality improvement and adaptive management decision-making processes. Also, this study provided basis that substantiated GHPP VaccTrains's multi-component approach to capacity development, which addresses structural deficiencies, staff scientific and regulatory training and support for regional networks (e.g., AVAREF) in a concerted manner. Indeed, this is what may likely be worthwhile to retain and sustain capacity development gains and produce the desired long-term outcomes and ultimate impact.

In summary, from this monitoring and evaluation study, we here provide a practical illustration establishing that upstream technical structures for CTO (and perhaps fellow affiliation/selection) are fundamental to achieving capacity development short- and long-term outcomes. More also, we show that certain external factors to the CT regulatory structure, such as vibrancy of CT activity, may influence the success of a capacity development intervention, but only early in the implementation phase. Furthermore, we show that internal NMRA mechanisms for setting and specifying expectations to beneficiaries of external trainings could be a useful catalyst to in-house implementation and sharing of newly gained knowledge. We encourage that other capacity development programmes monitor and evaluate the effectiveness of their interventional efforts in the early cycles of similar projects in order to optimize strategies in response to various challenging situations. This will ensure that their interventions achieve the set goals and promote sustainability in recipient NMRAs.

## Data Availability Statement

The datasets generated and/or analyzed during the current study are not publicly available due to confidentiality and data protection rights but are available from the corresponding author on reasonable request.

## Ethics Statement

Ethical review and approval was not required for the study on human participants in accordance with the local legislation and institutional requirements. The patients/participants provided their written informed consent to participate in this study.

## Author Contributions

SO: study concept and design, acquisition of data, analysis and interpretation of data, visualization, drafting of the original manuscript, critical revision of the manuscript for important intellectual content, and final approval of manuscript. IŠ-Y: study concept and design, acquisition of data, analysis and interpretation of data, critical revision of the manuscript for important intellectual content, and final approval of manuscript. AP: acquisition of data, analysis and interpretation of data, critical revision of the manuscript for important intellectual content, and final approval of manuscript. HM: study concept and design, analysis and interpretation of data, critical revision of the manuscript for important intellectual content, final approval of manuscript, and funding acquisition. CC: study supervisor, study concept and design, analysis and interpretation of data, critical revision of the manuscript for important intellectual content, final approval of manuscript, and funding acquisition. All authors contributed to the article and approved the submitted version.

## Funding

This work was supported by funds provided by the German Federal Ministry of Health's Global Health Protection Programme and allotted to the RegTrain-VaccTrain project based on a decision by the German Bundestag (Grant Project Number: 323-123002).

## Conflict of Interest

The authors declare that the research was conducted in the absence of any commercial or financial relationships that could be construed as a potential conflict of interest.

## Publisher's Note

All claims expressed in this article are solely those of the authors and do not necessarily represent those of their affiliated organizations, or those of the publisher, the editors and the reviewers. Any product that may be evaluated in this article, or claim that may be made by its manufacturer, is not guaranteed or endorsed by the publisher.
