# Supplementary material for: The Pivotal Role of Quality Technical Structures for Clinical Trials Oversight in the Achievement of Long-Term Capacity Strengthening Outcomes
Source: Front Med (Lausanne). 2022 Mar 2;9:772605. doi: 10.3389/fmed.2022.772605 (PMC8924119; doi:10.3389/fmed.2022.772605)
Supplement: Supplementary file 1 [file Data_Sheet_1.PDF]

Supplementary Material

1 Supplementary Table 1: Assessment of respective NMRA CT guidelines at the short-term timepoint according to the WHO GBT for Clinical Trial Oversight

- **CTA Procedure, Processing and Review**

	Indicator	A	B	C	D	E	F	G	H	I
1	Guidelines that define the format and content of protocol, the procedure for submission, and the timeframe for review of application	Y	Y	Y	Y	P	P	Y	P	na
2	• Documentation specifying the format and nature of CT application submission package	Y	Y	Y	Y	P	P	Y	P	na
	• List of critical documents that should be included in the application package.	Y	Y	Y	Y	Y	Y	Y	Y	na
3	Guidelines that establish the criteria for reviewing CT applications.	Y	N	N	N	N	N	N	N	na
4	Guidelines that specify the detailed criteria to be used for evaluating CT applications.	Y	N	Y	P	N	P	P	P	na
5	Guidelines requiring the establishment and maintenance of a register, list or database of approved or rejected CT applications	P	N	P	P	N	N	N	N	na
6	• Guidelines that provide the list of situations in which the routine CT procedures may not be required.	Y	N	N	Y	N	N	N	N	na
	• Guidelines describing the content and format of CT applications requesting application of non-routine CT procedures such as fast-track.	P	N	N	Y	N	N	N	N	na
	• Guidelines specifying the scope of the evaluation process (i.e., screening, verification, etc).	Y	N	N	Y	N	N	N	N	na

- **GCP Inspections**

1	The current published and easily accessible GCP requirements for all CTs.	Y	Y	Y	Y	Y	Y	Y	Y	na
2	(i) Guidelines and (ii) checklists used to provide guidance in the GCP inspection process	Y	Y	Y	P	N	Y	N	Y	na

- **Detecting and Reporting Adverse Drug Reactions**

1	• Guidelines on monitoring and reporting of adverse events and reactions (AE&R), as well as the guidance on required follow up.	Y	Y	Y	Y	P	Y	P	Y	na
	• Guidelines on the procedures for monitoring and reporting of AE&Rs as in line with WHO or other international standards.	Y	Y	Y	Y	Y	Y	Y	Y	na
2	Guidelines detailing the established committee responsible for reviewing reports of AE&R, and documentation defining the composition of the committee and specialties of its members.	P	N	P	N	N	N	N	N	na
3	Guidelines defining the timelines allocated for reporting AE&R on the part of the investigator or sponsor and timelines for generating and submitting a report on the AE&R to the NRA.	Y	P	Y	P	P	Y	P	Y	na

Y: yes; N: no; P: partial; na: not applicable

2 **Supplementary Table 2:** Assessment of respective NMRA CT guidelines at the long-term timepoint according to the WHO GBT for Clinical Trial Oversight

- **CTA Procedure, Processing and Review**

Indicator		A	B	C	D	E	F	G	H	I
1	Guidelines that define the format and content of protocol, the procedure for submission, and the timeframe for review of application	Y	Y	Y	Y	P	Y	Y	Y	Y
2	• Documentation specifying the format and nature of CT application submission package	Y	Y	Y	Y	P	Y	Y	Y	Y
	• List of critical documents that should be included in the application package.	Y	Y	Y	Y	Y	Y	Y	Y	Y
3	Guidelines that establish the criteria for reviewing CT applications.	Y	N	N	N	N	Y	Y	Y	Y
4	Guidelines that specify the detailed criteria to be used for evaluating CT applications.	Y	Y	Y	P	N	P	P	P	P
5	Guidelines requiring the establishment and maintenance of a register, list or database of approved or rejected CT applications	P	Y	P	P	N	Y	Y	Y	Y
6	• Guidelines that provide the list of situations in which the routine CT procedures may not be required.	Y	P	N	Y	N	Y	Y	Y	Y
	• Guidelines describing the content and format of CT applications requesting application of non-routine CT procedures such as fast-track.	P	Y	N	Y	N	Y	Y	Y	Y
	• Guidelines specifying the scope of the evaluation process (i.e., screening, verification, etc).	Y	Y	N	Y	N	Y	Y	Y	Y

- **GCP Inspections**

1	The current published and easily accessible GCP requirements for all CTs.	Y	Y	Y	Y	Y	Y	Y	Y	Y
2	(i) Guidelines and (ii) checklists used to provide guidance in the GCP inspection process	Y	Y	Y	P	N	Y	N	Y	P

- **Detecting and Reporting Adverse Drug Reactions**

1	• Guidelines on monitoring and reporting of adverse events and reactions (AE&R), as well as the guidance on required follow up.	Y	Y	Y	Y	P	Y	Y	Y	Y
	• Guidelines on the procedures for monitoring and reporting of AE&Rs as in line with WHO or other international standards.	Y	Y	Y	Y	Y	Y	Y	Y	Y
2	Guidelines detailing the established committee responsible for reviewing reports of AE&R, and documentation defining the composition of the committee and specialties of its members.	P	Y	P	N	N	Y	P	Y	N
3	Guidelines defining the timelines allocated for reporting AE&R on the part of the investigator or sponsor and timelines for generating and submitting a report on the AE&R to the NRA.	Y	Y	Y	P	P	Y	Y	Y	Y

Y: yes; N: no; P: partial; na: not applicable

Supplementary Figure 1: Long-term follow-up evaluation of fellows' outcomes and improvements of CTO activity at the NMRA level

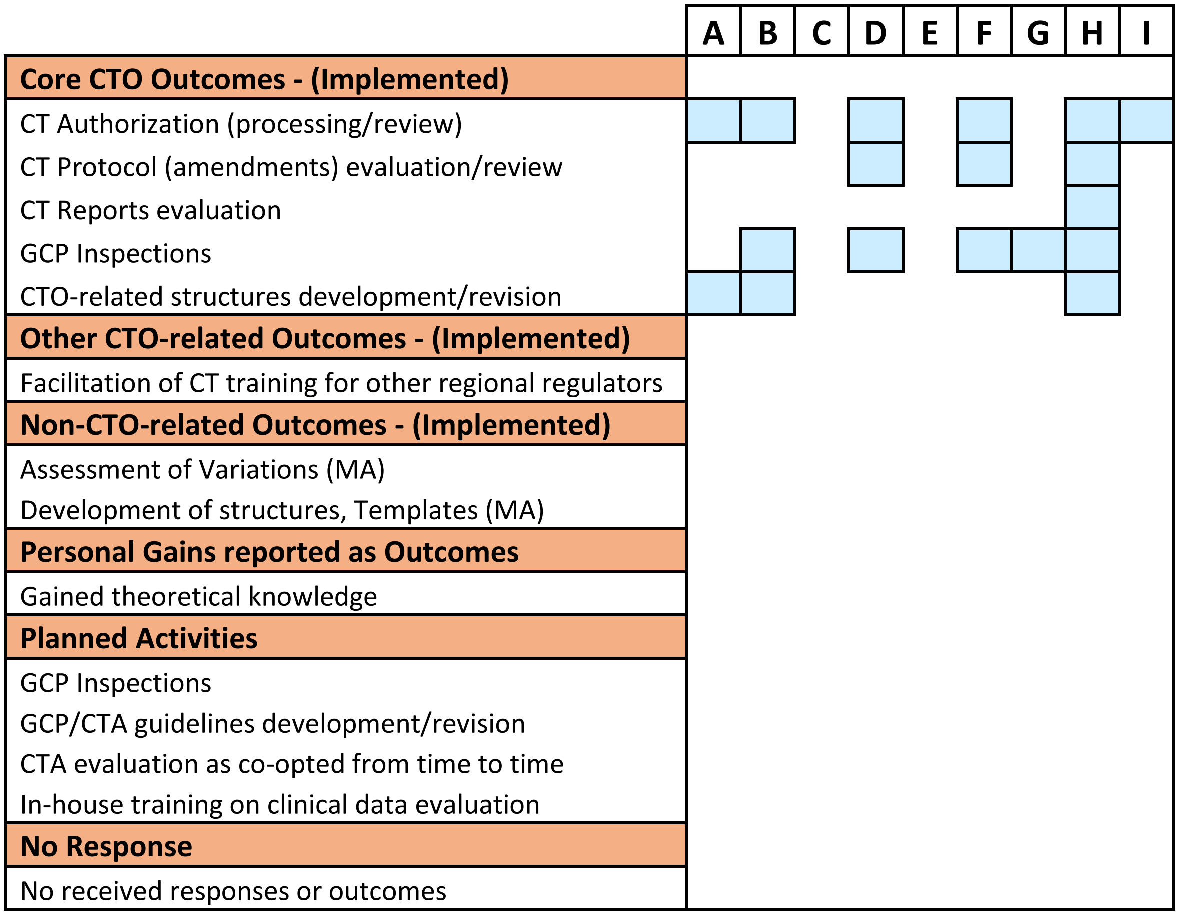

Information was sought from fellows using an electronic survey on issues of achieved training outcomes and organizational-level improvements in CTO 15 months after the training. This figure illustrates the individual responses from all sponsored fellows showing the kind of reported outcomes (CTO-related, non-CTO-related, others).
